# Light-Responsive Nanocapsule-Coated Polymer Films for Antimicrobial Active Packaging

**DOI:** 10.3390/polym11010068

**Published:** 2019-01-05

**Authors:** Valentina Marturano, Valentina Bizzarro, Veronica Ambrogi, Adele Cutignano, Giuseppina Tommonaro, Gennaro Roberto Abbamondi, Marta Giamberini, Bartosz Tylkowski, Cosimo Carfagna, Pierfrancesco Cerruti

**Affiliations:** 1 Institute for Polymers, Composites and Biomaterials (IPCB-CNR) Via Campi Flegrei, 34, 80078 Pozzuoli (NA), Italy; v.bizzarro@alice.it (V.B.); carfagna@ipcb.cnr.it (C.C.); 2 Department of Chemical, Materials and Production Engineering (DICMAPI) University of Naples “Federico II”, P. le Tecchio, 80, 80125 Napoli, Italy; ambrogi@unina.it; 3 Institute of Biomolecular Chemistry (ICB-CNR) Via Campi Flegrei, 34, 80078 Pozzuoli (NA), Italy; acutignano@icb.cnr.it (A.C.); gtommonaro@icb.cnr.it (G.T.); gennaroroberto.abbamondi@icb.cnr.it (G.R.A.); 4 Department of Chemical Engineering (DEQ), Universitat Rovira i Virgili, Av. Països Catalans, 26, 43007 Tarragona, Spain; marta.giamberini@urv.cat; 5 Chemistry Technology Centre of Catalonia (CTQC), C/Marcel·lí Domingo, 43007 Tarragona, Spain; bartosz.tylkowski@ctqc.org; 6 Institute for Polymers, Composites and Biomaterials (IPCB-CNR) Via Previati 1/C, 23900 Lecco, Italy; cerruti@ipcb.cnr.it

**Keywords:** active packaging, photo-responsive nanocapsules, essential oils, antimicrobials, coatings

## Abstract

The development of antimicrobial active packaging constitutes a powerful tool to reduce waste and increase quality standards of perishable goods. Among numerous available antimicrobial agents, essential oils stand out for their renowned efficiency, and their use is beneficial due to their sustainability compared to other oil-based antimicrobials. In this work, we report on the use of photo-responsive nanocapsules containing thyme essential oil as functional coatings for polyethylene and polylactic acid films to obtain antimicrobial active packaging. Polymer surface activation treatment enhanced compatibility with nanocapsules solution. The films were analyzed to assess the structural and functional properties of the coating, evaluate morphological changes due to their photo-responsive behavior, and monitor the light-induced release of volatile thyme oil. It was found that 24 h after a 15-min UV exposure of the coated films, the concentration of thyme oil in the headspace was eight times higher with respect to un-irradiated films, thus confirming the efficiency of the light-triggered release system. Therefore, the manufactured films are proposed as on-demand release devices for application in non-contact antimicrobial active packaging.

## 1. Introduction

Modern society, where consumer goods are readily available, is increasingly inclined to favor waste, especially of food. Over the last few decades, the necessity to address feeding issues related to increasing world population has pushed scientific efforts toward greater awareness in the food industry [1,2]. Materials technology can help minimize food losses and improve quality, freshness, and safety of food products. In fact, a wide variety of “active” packaging technologies have been developed to meet these requirements. Important examples of active packaging include oxygen scavengers, carbon dioxide emitters/absorbers, moisture absorbers, ethylene absorbers, ethanol emitters, flavor releasing/absorbing systems, time-temperature indicators, and antimicrobial-containing films [3]. Active, or smart, packaging has often been described as any kind of packaging that provides specific functionality beyond the role of a physical barrier between the food product and the surrounding environment [4]. 

**Figure 1:**
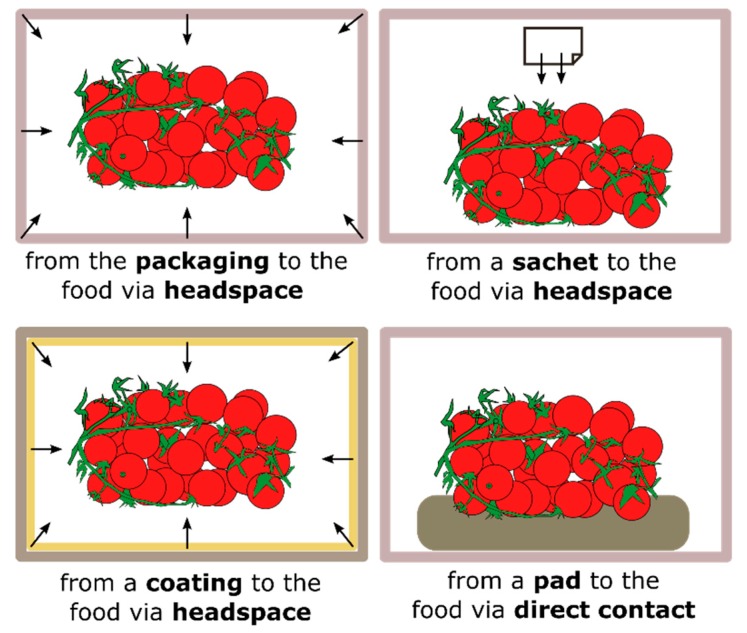
Methods for the release of antimicrobial agents in food packaging technologies.

More recently, a novel concept of packaging has been introduced: responsive packaging has been defined as any packaging that elicits a response as a result of a specific trigger or change occurring in the food product, food package headspace, or the outside environment [5]. One of the most interesting approaches to responsive packaging involves the use of intelligent nanosized carriers employed as either coating or filler for the packaging material [6]. Stimuli-responsive nanocarriers are able to encapsulate an active material, facilitate its manipulation, and protect it from the outer environment, while also enabling its on-demand release by application of an external stimulus (temperature, magnetic field, pressure, light) [7,8,9]. The use of such systems has been particularly investigated in the field of antimicrobial (AM) packaging, where antimicrobials are incorporated into the packaging to prevent bacterial growth, reduce the risk of pathogen contamination, and extend the shelf life of minimally processed foods [10]. Different functioning mechanisms of AM food packaging are shown in Figure 1. For instance, AM agents can diffuse through the headspace in a non-contact mode or migrate to the food by a direct contact between the packaging material and the food. For some food products, such as fresh meat, in which the microbial contamination occurs primarily at the surface, direct surface application of AM agents has limited benefits, because they rapidly migrate into the food mass. On the other hand, the non-contact and prolonged release of AM agents from packaging, coatings or sachets can help maintain high AM concentrations when needed [11,12]. However, food application of an antimicrobial packaging system is limited due to the availability of suitable antimicrobials, new polymer materials, regulatory concerns, and appropriate testing methods [13]. 

Among the most investigated antimicrobial agents for AM packaging, essential oils (EO) stand out for their efficient bactericide activity and their availability [14,15]. EOs extracted from plant materials are also viewed as a more sustainable alternative to synthetic antimicrobial agents. However, incorporation of EOs in polymer films can result in physical changes of the matrix due to the polymer–EO components interactions. As a consequence, negative impacts on film structure, barrier properties, and transparency have been reported [16]. Moreover, large amounts of EOs must be introduced in the polymer to elicit the AM activity. Therefore, the coating of commercially available films with a thin layer of active agent is an efficient and cost-effective approach, one that is increasingly adopted in packaging science and technology [17,18]. When used in polymer coating formulations, EOs should be encapsulated in order to protect them from chemical degradation and premature evaporation. Today, a wide range of encapsulation systems are being proposed, such as hydrogels [19], liposomes [20], cyclodextrin [21], and polymer micro- and nano-capsules [22,23]. 

In this work, we present a responsive packaging system obtained by coating commercial packaging films, such as polyethylene (PE) and polylactic acid (PLA), with photo-responsive nanocapsules (NCs) containing antimicrobial thyme essential oil. The AM properties of thyme EO are universally recognized; moreover, its highly volatile components allow its use in non-contact packaging applications [24]. The photo-responsive polymeric NCs were prepared via interfacial polycondensation in miniemulsion, as reported in our previous work [25]. UV light (λ_max_ = 360 nm), a wavelength present even in the sun emission spectrum, was used to induce the E-Z photo-isomerization in the azobenzene segments embedded in the capsule shell. The presence of the photo-sensitive moiety induced a conformational rearrangement of the polymeric chains of the capsule shell upon UV irradiation, triggering the release of the encapsulated EO. Therefore, the manufactured films are proposed as on-demand release devices for application in non-contact antimicrobial active packaging.

## 2. Materials and Methods 

### 2.1. Materials

An unstabilized grade of a butene copolymer linear low-density polyethylene (PE), DJM1826, with a melt flow index (MFI) of 2.5 g 10 min^−1^ was supplied as a powder by Versalis (Milano, Italy). Polylactic acid (PLA) grade 4042D (94% l-lactic acid) was obtained from NatureWorks, LLC (Minnetonka, MI, USA). Thyme EOs were purchased from Farmalabor, (Canosa di Puglia, Italy) and used without further purification. Polymer NCs containing thyme EO were synthesized as reported elsewhere [23] and coded as NCT. 

### 2.2. Evaluation of Thyme EO Antimicrobial Activity

In order to determine the optimal amount of thyme oil needed to manufacture the NC-coated polymer films to be applied in AM packaging applications, the inhibitory effect of thyme oil against two model bacterial strains was evaluated. The antimicrobial activity was tested using liquid cultures of *Escherichia coli* (DSM 498) and *Micrococcus luteus* (DSM 348) grown in nutrient broth (Oxoid) for 24 h at 37 °C. The Minimum Inhibitory Concentration (MIC) was determined by a serial dilution, in triplicate, from 10 μg/mL to 0.01 μg/mL. Microbial growth was determined after 24 and 48 h of incubation by spectrophotometric measurement with a Microplate Reader (Tecan Genios Pro, Mannedorf, Switzerland) that was equipped with a 540 nm filter. 

### 2.3. Preparation and Surface Treatments of PE and PLA Films

PLA pellets were oven-dried for 24 h at 65 °C under vacuum, prior to extrusion. PLA films were prepared using a Collin E 20T single screw extruder equipped with a Collin CR 72T calendaring unit, using the following temperature profile (from hopper to die): 165, 170, 170, 170, and 170 °C. The thickness of the obtained film was on average 60 ± 10 μm. PE films were obtained using the same equipment, with the following temperature profile: 150, 170, 180, 180, and 170 °C. PE film thickness was 68 ± 12 μm.

Both PE and PLA films were then subjected to surface treatments (corona and plasma) to increase their wettability and promote the adhesion of the nanocapsules coating. Plasma treatment was performed on square polymer films (surface area of 2 cm^2^) employing a Tucano medium plasma reactor 600 (Gambetti Kenologia, Milano, Italy) for 12 min. Alternatively, corona treatment was performed on other polymer films employing a Tantec corona effect machine at 150 W for 10 min. In order to verify the efficiency of the two surface treatments, contact angle measurements were performed using an OCA/Dataphysics/SCA20 Contact Angle System (Filderstadt, Germany). In a typical experiment, 2 µL of water or 1 µL of diiodomethane are deposited on the surface of the film under study, and the contact angles formed by the droplets of the two liquids on the substrate are then measured by optical microscopy. This test was performed to select the best surface-activation method for each specific polymeric substrate, to maximize the adhesion in the coating step. 

### 2.4. Coating of PE and PLA Films with NCT

Prior to deposition on the polymeric films, NCT, or polymer NCs containing thyme EO or, underwent a two-step purification process. First, 20 mL of the NCT aqueous solution were centrifuged at 9000 rpm for 30 min at 4 °C in order to separate the capsules from the Mowiol 18-88 surfactant present in the water phase. NCT, obtained as a slurry precipitate, were dispersed in 20 mL of milliQ water. Phase extraction was then performed using a 1:1 *v/v* proportion of aqueous NCT/hexane in order to remove the free EO still present in the NCs solution. The coating of plasma-activated PE and corona-activated PLA substrates with NCT suspensions was performed by depositing 2 mL of the purified NCT solution on a 20 cm × 30 cm PLA or PE sheet using a bar coater to create a uniform layer. Prior to characterization, the coated polymer films were dried for 12 h at room temperature. 

Surface morphology of nanocapsule-coated films was observed via scanning electron microscopy (SEM) using a FEI Quanta 200 FEG instrument (Eindhoven, Netherlands) in high vacuum mode, equipped with a Large Field Detector operating at acceleration voltages ranging from 15 to 20 kV. For SEM sample preparation, a 25 mm^2^ film specimen was placed on an aluminum stab and metallized with an Au-Pd coating. The most efficiently coated system was then selected for further characterization. Adhesion tests were performed on the coated films by slight modification of a procedure reported in literature [26]. A 4 × 4 cm^2^ coated film sample was secured with adhesive tape on a 10× Axioscop Zeiss microscope stage with the coated surface facing upward. While the film remained in place, adhesive tape was applied to the coated surface and firmly rubbed to ensure good contact between the tape and the film. After 90 s the tape was pulled with a steady motion at a 180° angle. Optical micrographs with a 10× magnification were snapped before and after the test. A 10 × 10 grid of 1 µm × 1 µm squares was superimposed to the images and in each square was evaluated to monitor adhesion.

In order to evaluate the photo-responsive activity of the NC system, the coated films were characterized before and after irradiation via UV–Vis and FTIR spectroscopy using a V570 UV–Vis Jasco spectrophotomer, and a Spectrum 100 Perkin–Elmer FTIR spectrophotometer equipped with a single bounce diamond ATR device, acquiring 16 scans at 4 cm^−1^ resolution. 

### 2.5. Release of EO from NCT-Coated Films

Thyme EO is composed of a mixture of volatile components, therefore gas chromatography–mass spectrometry (GC–MS) was used to evaluate the release kinetics of the oil from the film upon UV irradiation. To perform these measurements, a 20 × 30 cm^2^ NCT-coated PE film was prepared by deposition of 2 mL of NCT aqueous solution (oil concentration 0.3 mg/cm^2^) on an activated PE thin film. The film was then left to dry for 15 h under a hood at room temperature. Then a 10 cm^2^ piece of film was cut and introduced in a quartz testing tube that was sealed with a gas-tight stopper with a pierceable septum. The sample was kept at a constant temperature of 25 °C and 0.5 mL of the headspace was collected and introduced in the GC–MS equipment to be analyzed. 

GC–MS runs were performed on a Focus GC-Polaris Q gas chromatography-mass spectrometer (Thermo Fisher Scientific, Waltham, MA, USA) equipped with a capillary column 5% diphenyl/95% dimethyl polysiloxane (30 m × 0.25 mm ID × 0.25 µm film thickness, Thermo Fisher Scientific) and an electron ionization (EI) source. The temperature program was set as follows: Initial 60 °C holding for 3 min, then heating to 150 at 2 °C/min, followed by an increase up to 310 °C at 20 °C/min, holding for 3 min. Inlet temperature: 210 °C; ion source: 250 °C; transfer line: 250 °C; He flow: 1 mL/min. Mass range was 50–400 m/z. To evaluate the EO release, the ion current associated with thymol molecular ion eluting at RT = 27.96 min was monitored. Absolute values of peak area (arbitrary units) corresponding to 0.5 mL gas phase injection were reported as a function of time of sampling. The sample was tested at time 0 and, after a 15-min irradiation with UV light at 365 nm, then at *t* = 3 and 24 h. The EO release from a non-irradiated sample was also evaluated as a control. Experiments were repeated in duplicate. 

## 3. Results and Discussion

The purpose of this work was to obtain a performing stimuli-responsive packaging capable of releasing incorporated antimicrobial active agents to increase the shelf life of the packaged material. To this aim, the use of light-responsive NCs is an attractive option for the triggered delivery of active compounds, since light is remotely controllable and available at virtually no cost. As demonstrated in a previous work [27], natural essential oils can be efficiently encapsulated in smart polyamide nanocapsule obtained via interfacial polycondensation in miniemulsion. Their photo-responsive behavior, achieved by means of photochromic azobenzene segments embedded in the capsule shell, is triggered by UV light irradiation (λ_max_ = 360 nm). 

**Figure 2:**
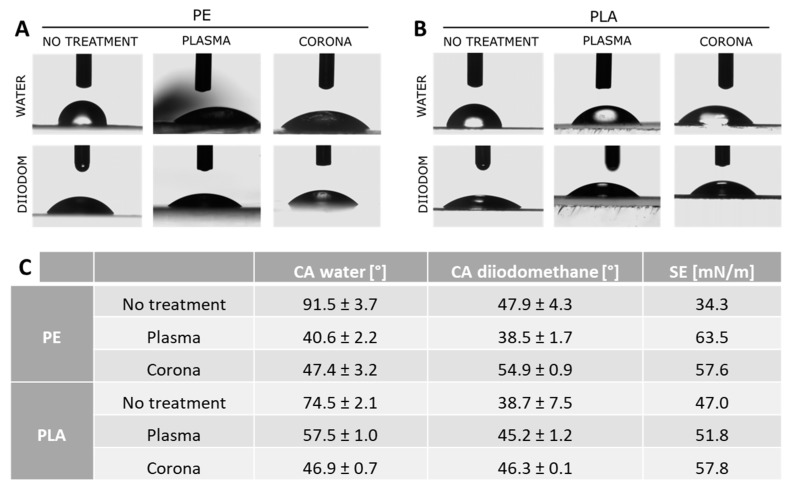
Contact angle micrographs performed on untreated and plasma and corona-treated (**A**) polyethylene (PE) and (**B**) polylactic acid (PLA). (**C**) Contact angles and surface energy values calculated from contact angle measurements using water or diiodomethane as reference liquids.

The main problem related to the coating process was the inherent incompatibility between the hydrophobic polymer substrate and the aqueous NCs suspension. To facilitate and optimize the deposition of the NCs functional layer, the selected packaging materials were subject to surface treatment (plasma and corona) to improve their wettability prior to the coating step. Therefore, to assess and compare the effects of treatments on PE and PLA surface properties, contact angle measurements were carried out. The smaller the contact angle, the higher the affinity of the surface is for the reference liquids, resulting in a higher wettability. In Figure 2, the contact angle measurements for water and diiodomethane on PE (Figure 2A) and PLA (Figure 2B) films are reported along with the associated values of surface energy (SE) (Figure 2C). Depending on the surface treatment applied, a different response was observed for the two analyzed materials. In particular, PE films exhibited higher wettability upon plasma treatment, while in the biodegradable polymer PLA film, corona treatment was more effective. Therefore, plasma-treated PE and corona-treated PLA were selected for further investigation.

PE and PLA films were coated with NCT as reported in Section 2.3. In order to determine the amount of NCT to apply on the polymer substrates to manufacture antimicrobial films, the Minimum Inhibitory Concentration (MIC) of thyme oil against two model bacterial strains, namely *Escherichia coli* (DSM 498) and *Micrococcus luteus* (DSM 348), was evaluated. 

**Figure 3:**
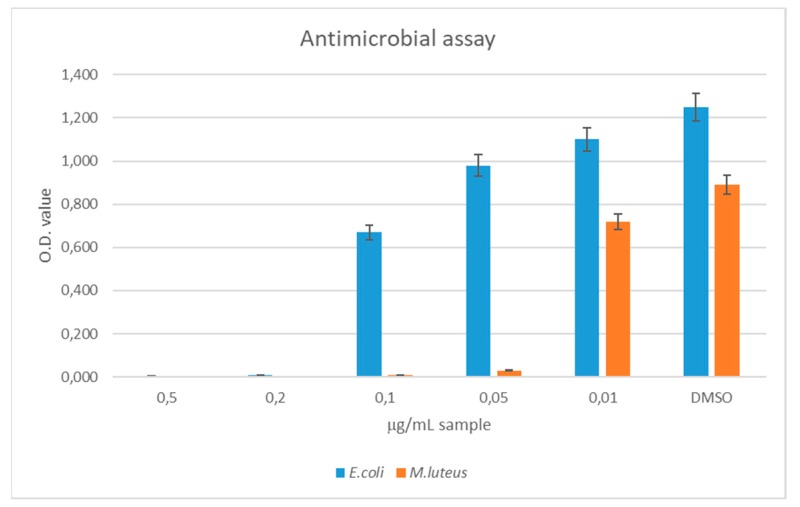
Antimicrobial activity of thyme essential oil against *E. coli* (**blue**) and *M. luteus* (**orange**).

To this aim, the oil was introduced into the culture medium and the optical density was measured at different concentrations. Results reported in Figure 3 show that the minimal inhibitory concentration of thyme oil was 0.2 µg/mL for *E. coli* and 0.05 µg/mL for *M. luteus*. This experiment’s results served as a guideline for the preparation of NC-coated films. Considering that 0.2 µg/mL of thyme oil was able to inhibit microbial growth of both microorganisms, a standard volume of 100 mL would be efficiently preserved using 20 µg or 0.022 µL (*d* = 0.9 g/mL) of thyme oil. Since the oil phase made up 1/7 of the total liquid phase in the NCs preparation miniemulsion, and the encapsulation efficiency was calculated to be 95%, 0.16 µL of NCT solution was needed to efficiently preserve 100 mL of perishable product. 

This value refers to the volume of antimicrobial agent injected into the liquid medium. However, for non-contact application, NCT-coated polymer films should release the encapsulated EO in vapor phase upon UV-light irradiation. For this reason, a 600 cm^2^ film was coated with a 2 mL volume of NCT, in order to provide a far greater amount of oil available in the packaging headspace. 

**Figure 4:**
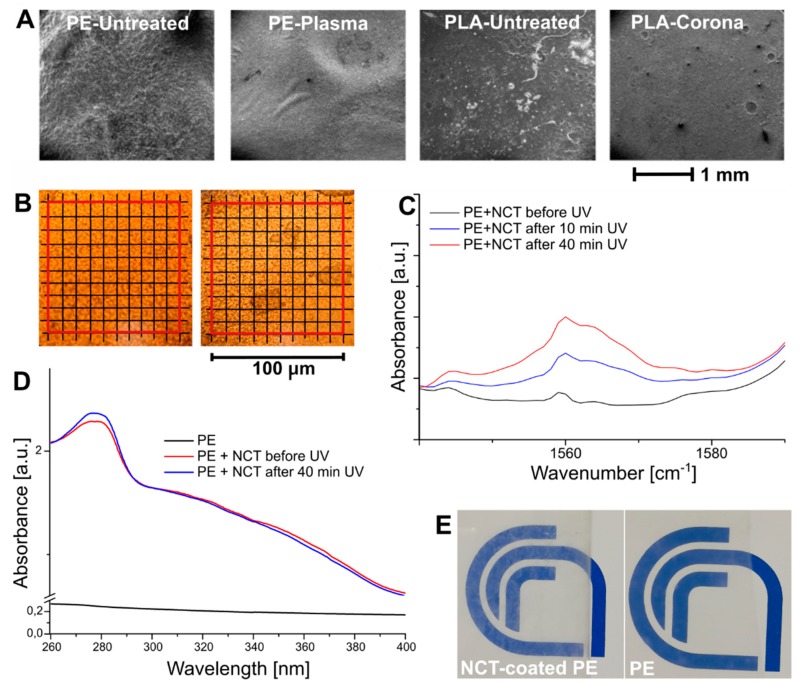
(**A**) SEM micrographs of treated PE and PLA films coated with polymer NCs containing thyme EO (NCT), (**B**) optical micrographs of PE coated with NCT before and after the adhesion test, (**C**) FTIR and (**D**) UV–Vis spectra of PE coated with NCT before and after UV irradiation at 366 nm, and (**E**) optical images showing the transparency of PE and NCT-coated PE.

NCT-coated PE and PLA films were produced, and the coating’s morphological and adhesion features were analyzed. An efficient deposition process resulted in a more uniform layer of EO-loaded capsules, thus providing a greater surface area for the release of the encapsulated essential oil. SEM micrographs of NCT-coated PE and PLA films treated with plasma and corona, respectively, compared with untreated NCT-coated polymer films are reported in Figure 4A. As expected, untreated polymers exhibited poor compatibility with nanocapsules suspensions, as evidenced by the appearance of inhomogeneous areas, which were particularly evident in PLA samples. As mentioned above, plasma treatment improved PE wettability, providing uniform coatings upon deposition of NCT suspension. In the case of corona-treated PLA, comparable results were obtained from SEM analysis, although some impurities were still identified on the coated surface (Figure 4A). Consequently, NCT-coated PE samples were selected as representative for the subsequent analysis on the release behavior. In Figure 4B, optical microscopy images before and after the adhesion tests are shown. The surface of the coated film before the test, as can be seen from the images, appeared homogeneous, while about 17% of the film surface showed a coating detachment upon tape removal, indicating a strong adhesion between NCT and the treated-PE substrate. 

The E-Z isomerization is a key parameter for governing the properties of photoswitchable molecules. The isomerization pathways and kinetic quantities of azobenzene have been elucidated by theoretical methods [28,29], however, their experimental determination is not straightforward, due to the surrounding environment of the azobenzene moiety [30]. Photoswitchable molecules typically do not change their conformation in the pure crystalline state, while the conformation change in solution is affected by the solvent viscosity and polarity [31]. When embedded in polymers, the conformational change is strongly affected by the polymer matrix, and the efficiency of the process can be reduced, resulting in small changes in the spectroscopic properties of the two isomers [32,33]. In the present work, we used FTIR and UV–Vis spectroscopy to characterize the NCT-coated PE film, before and after UV exposure, to gather information on the occurrence of the isomerization of the azobenzene segments in the polymeric NCs shell. 

Figure 4C shows the FTIR spectra of NCT-coated PE film before and after 10 and 40 min of UV irradiation, in the 1540–1590 cm^−1^ wavenumber range. The vibrational peak of the Z isomer at 1560 cm^−1^, attributed to the ν(C=C) stretching vibrations of the phenyl rings became more intense as the UV exposure time increased [34]. UV–Vis analysis confirmed the occurrence of the E-Z isomerization. Figure 4D shows a slight decrease in the 340–380 nm absorbance range upon irradiation, due to the disappearance of the high-intensity π→π* absorption of the E azobenzene conformer. Correspondingly, an increase of the absorption peak at 280 nm, attributed to the phenol rings of thyme oil, was noticed due to the light-induced release of the EO [25]. Moreover, a significative increase in the absorbance of the NCT-coated PE, with respect to pristine PE, was observed. This outcome is macroscopically visible in Figure 4E, where the transparent PE becomes slightly more opaque when coated with NCT. 

**Figure 5:**
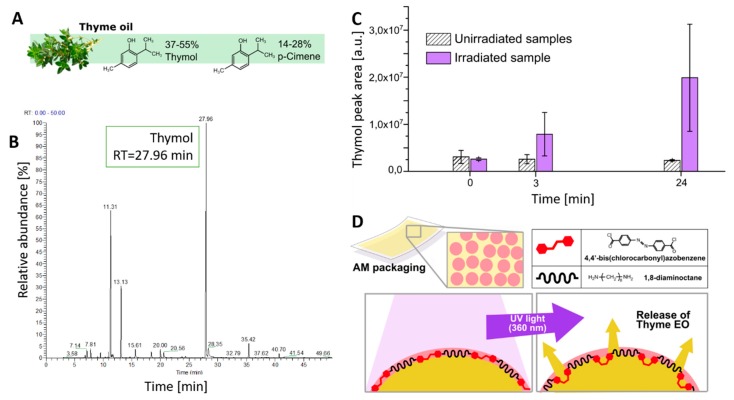
(**A**) Thyme oil major volatile components, (**B**) GC-MS chromatogram of thyme oil highlighting the presence of thymol as a major peak at RT = 28 min, (**C**) evolution of the thymol release evaluated by GC–MS for un-irradiated and UV-irradiated NCT-coated PE film, (**D**) NCT-coated nanocapsules releasing mechanism.

As a typical EO, thyme oil is a mixture of volatile molecules such as thymol (37–55%), *p*-cimene (14–28%), as shown in Figure 5A, and other compounds present at lower concentrations (γ-terpinene 4–12%, linalool 1.5–6.5%, carvacrol 0.5–5%, and β-myrcene 1–3%). GC–MS was employed to determine the relative abundance and retention time of different components of thyme oil. Results, reported in Figure 5B, indicated that the most abundant peak, attributed to thymol, had a retention time of about 28 min. The release of volatile thymol contained in thyme EO was evaluated by GC–MS measurements on NCT-coated PE films. For this purpose, two NCT-coated PE films were placed in two 15 mL quartz testing tubes; one was kept in darkness, while the other was irradiated for 15 min with a UV lamp (360 nm) to trigger the EO release from the photo-responsive NC coating. The headspace of each tube was withdrawn with a gas syringe and analyzed by GC–MS. The results, reported in Figure 5C, show that prior to irradiation (*t* = 0) the thymol concentration in both samples was comparable. Three hours after irradiation, the thymol concentration increased in the irradiated sample, while it remained constant in the non-irradiated one, proving the reliability of the release mechanism. Twenty-four hours after irradiation, the mean thymol concentration increased about 8 times with respect to the initial value. This result confirms the efficiency of the UV-triggered release schematized in Figure 5D.

## 4. Conclusions

Thyme EO is a well-known antimicrobial agent, used in many food packaging applications. In the present paper, we report the preparation and characterization of nanocapsule-coated polymer films able to release thyme EO under UV light exposure for active packaging applications. The coating process of PE and PLA with thyme EO-loaded capsules was carefully assessed, determining that a surface treatment, namely plasma or corona activation, was able to increase the wettability of the polymer surface. The NCT-coated films were analyzed to evaluate the coating’s morphological and adhesion features. Plasma-treated PE exhibited higher wettability and coating homogeneity; moreover, the NC coating layer was strongly adhering to the treated-PE substrate. The films were characterized with UV–Vis and FTIR spectroscopy to evaluate their response to UV irradiation, and it was found that immobilization of the NCs onto the polymer surface did not hinder the E-Z isomerization. The release efficiency of the light-activated films was assessed by GC-MS analysis, and it was found that even a 15 min UV exposure resulted in an 8-fold concentration increase of thyme oil in the headspace after 24 h, thus confirming the efficiency of the light-triggered release system. The applications of this promising release technology go far beyond the food packaging industry, since its results are also appealing for drug delivery, agriculture, household, and cosmetics applications.
